# A guide to cell-type-specific Cre mouse models in atherosclerosis: from background strains to future directions

**DOI:** 10.3389/fcvm.2026.1905454

**Published:** 2026-09-18

**Authors:** Tianhan Li, Xin Dong, Xinyao Niu, Haoyue Zhang, Jianjun Wu, Liaoxun Lu, Yinming Liang, Lichen Zhang

**Affiliations:** 1 School of Basic Medicine, Henan Medical University, Xinxiang, China; 2 School of Medical Technology, Henan Medical University, Xinxiang, China; 3 Center of Disease Model and Immunology, Hunan Academy of Chinese Medicine, Changsha, China

**Keywords:** AAV-based approach, atherosclerosis, cre line, lesion-associated cells, mice

## Abstract

Atherosclerosis is a chronic vascular inflammatory disease, and mouse models are essential for studying its pathogenesis. The Cre/loxP technology has become the gold standard for cell-type-specific gene targeting. However, the rapid proliferation of Cre driver lines has made model selection increasingly challenging. In this review, we provide a practical guide to choosing appropriate Cre mouse models. We compare the two most common backgrounds (*Apoe* and *Ldlr* knockout) in terms of lipid profiles, inflammatory dynamics, dietary dependence, and suitability for studying metabolic comorbidities to guide the selection of background strains, and systematically evaluate Cre models targeting all major cell types in atherosclerotic plaques (endothelial cells, smooth muscle cells, macrophages, neutrophils, dendritic cells, T cells, B cells, fibroblasts, NKT cells, and platelets), as well as lipid metabolism, hematopoietic cells and emerging AAV-based approaches. We also compare conventional knockout with conditional and inducible systems, emphasizing critical considerations, including Cre toxicity, tamoxifen leakiness, and experimental controls. Finally, we discuss future directions such as dual-recombinase, lineage tracing, comorbidity modeling, single-cell technologies, and cross-species validation, to better align mouse models with human atherosclerosis. By clarifying how model choice influences experimental outcomes, this review aims to help researchers select optimal models and accelerate mechanistic and therapeutic discoveries.

## Graphical Abstract

**Figure:**
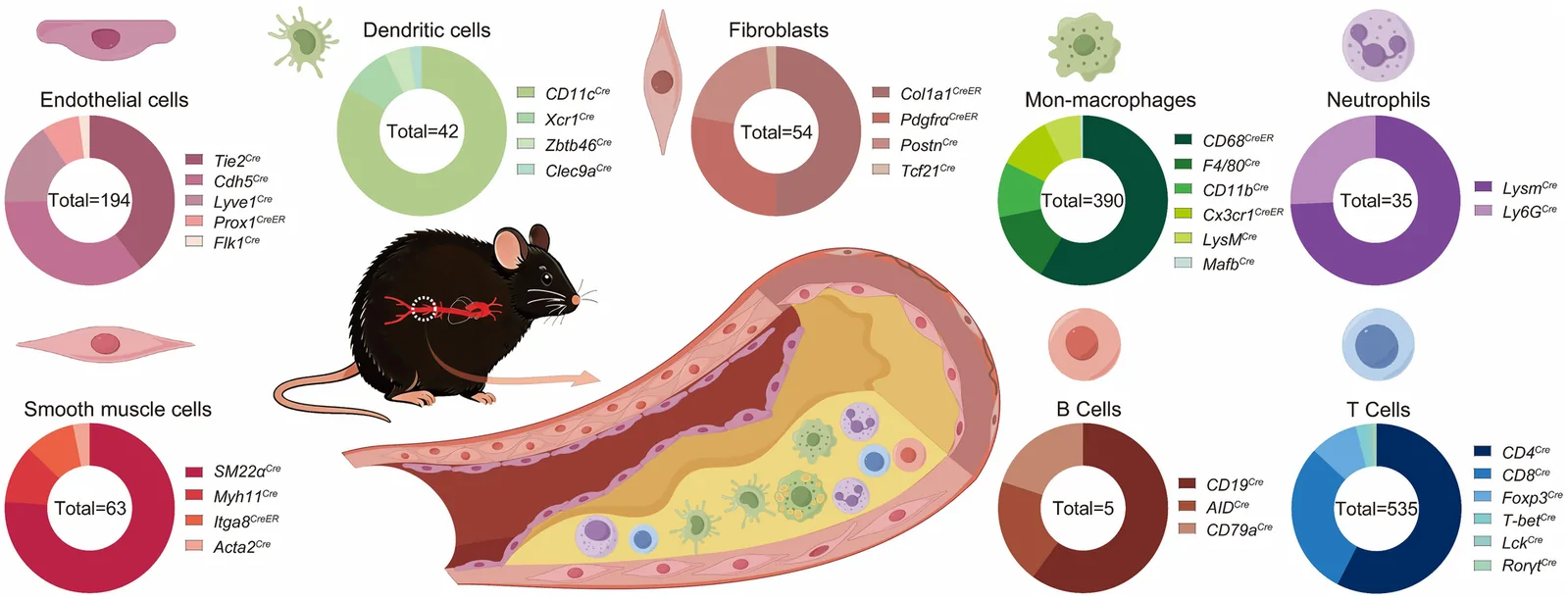
Schematic diagram of cells involved in atherosclerotic plaque formation and annual publication trends of different models of the respective cells. Notes. Cre/loxP transgenic mouse models for studying cellular contributions to atherosclerotic plaque formation. Donut charts show the number of publications (2021–2025) using Cre/loxP transgenic mouse models to study cell-specific contributions to atherosclerotic plaque formation. Each chart represents a distinct cell population, with the total number of publications labeled (“Total = N”) and segments corresponding to commonly used Cre driver lines. Cell types include endothelial cells, smooth muscle cells, fibroblasts, dendritic cells, monocyte-macrophages, neutrophils, B cells, and T cells. The schematic depicts the cellular composition of an atherosclerotic vessel cross-section. Publication counts were retrieved from the PubMed database for the period 2021–2025; Search strategies are detailed in the Supplementary Table S1.

## Highlights

- Systematic comparison of *Apoe* and *Ldlr* knockout backgrounds
- Critical evaluation of Cre models targeting ECs, SMCs, and immune cells
- Practical framework for selecting optimal mouse models
- Comparison of conventional KO vs. conditional and inducible Cre-loxP systems

## Introduction

Atherosclerosis and its clinical sequelae are the core of cardiovascular disease (CVD), including myocardial infractions, stroke, and peripheral vascular disease (1). A variety of animal models have been employed to study the atherogenic process, including chickens (2), pigeons (3) mouse (4), rats (5), rabbits (6), dogs (7), non-human primates (8), and zebrafish (9). Among them, mouse models are the most frequently used species for atherosclerosis studies due to its unique comprehensive advantages among model organisms (10). Mouse models not only share many genetic and biological similarities with humans (11), but also offer practical benefits, such as low cost, rapid and convenient breeding, high reproducibility, and a well-defined genetic background (12), along with mature gene editing technology. These features make mice the most important model animals for studying disease pathogenesis, establishing human-related disease models, and conducting preclinical drug evaluation (13). The use of experimental animal models in the drug development process helps to understand the origin, pathophysiological characteristics, mechanisms, drug target identification, properties, toxicity and safety, pharmacokinetics and efficacy evaluation, as both phenotype-based and targeted drug development rely heavily on animal models (14). In the early stages, evaluation of scientific hypotheses and potential drug treatments begins with the construction of mouse disease models and subsequent laboratory studies (15). The emergence and advancement of Clustered Regularly Interspaced Short Palindromic Repeats (CRISPR) editing technology have made it easier and more effective to construct gene-edited mouse disease models that simulate human-related diseases (16).

Beyond CRISPR-based approaches, the Cre (cyclization recombination)-loxP (locus of X-over P1) system, derived from bacteriophage P1, provides another powerful tool for conditional gene manipulation. The system consists of the Cre recombinase (38 kDa) and 34-bp loxP recognition sequences. The loxP sites are directional, and Cre mediates excision between directly oriented sites or inversion between oppositely oriented sites via a cleavage and religation mechanism without DNA double-strand breaks, resulting in low off-target risk (17). By inserting loxP sequences flanking critical exons of a target gene (generating a “floxed” allele) and combining this with tissue-specific or inducible Cre expression, spatiotemporal gene inactivation can be achieved (18). The molecular dynamics of recognition and catalysis in this system have been thoroughly characterized, while practical applications require careful attention to potential issues such as unintended recombination, ectopic Cre expression, and Cre associated toxicity (19, 20). Recent advances include orthogonal loxP variants and combinatorial strategies with other recombinases such as Flp and Dre, greatly expanding the flexibility for multiplexed genetic manipulations (21).

Various animal models, both small and large, have been employed to study the process of atherosclerosis (22). No single model is perfect, as each has its advantages and limitations in manipulating atherosclerosis and simulating human atherosclerosis or lipoprotein profiles (23). The widespread use of mouse models is primarily due to the relative ease of genetic manipulation and the comparatively short time frame for atherosclerosis development (24). While not all aspects of murine atherosclerosis align with human conditions, studies using mouse models have unveiled underlying biological processes and interactions (25). Given that different factors influence various stages of lesion development, the use of mouse models with the ability to turn on or delete proteins or cells in a tissue-specific and temporal manner holds significant value (26). More importantly, different cell-specific Cre mouse models can lead to distinct, and sometimes opposing effects on atherosclerotic lesion development. Thus, selecting the appropriate model for a given research question requires careful consideration. In this review, we briefly compare two classical mouse models, and emphasize the advantages and limitations of Cre models targeting all major cell types in atherosclerotic plaques, which help future researchers to choose the most appropriate mouse model based on their scientific purpose.

## Two classical mouse models

Mice have become the main animal model for studying atherosclerosis since the 1990s. Currently, the most commonly used atherosclerosis mouse models are apolipoprotein E (*Apoe*) knockout (accounting for approximately 70% of studies) and low density lipoprotein receptor (*Ldlr*) knockout (accounting for approximately 27%) (27).

### *Apoe* knockout mice

*Apoe* knockout mice were successfully established in 1992 and showed markedly elevated plasma total cholesterol (28). When maintained on a standard chow diet, these mice develop complex atherosclerotic lesions accompanied by severe hypercholesterolemia and impaired immune regulatory function (29). A major advantage of this model is that it does not require Western diet induction to induce significant atherosclerotic lesions in mice (30). However, the key limitation is that very low-density lipoprotein (VLDL), rather than low density lipoprotein (LDL), constitutes the main lipoprotein fraction in these mice, which differs from the human lipid profile characterized predominantly by LDL (31). Beyond its role in mediating lipoprotein clearance, *Apoe* itself possesses additional functions, including antioxidant, anti-proliferation, and anti-inflammatory activities (32). Consequently, the atherosclerotic lesions observed in *Apoe* knockout mice may not be solely attributed to alterations in plasma lipid levels (33).

### *Ldlr* knockout mice

The *Ldlr* knockout mouse model, first established in 1993, exhibits a moderate increase in plasma LDL cholesterol levels (34). While these mice develop only mild, late-onset atherosclerotic lesions on a standard chow diet, they are highly susceptible to diet-induced atherosclerosis. When fed a Western diet, they rapidly develop significant hypercholesterolemia and extensive atherosclerotic plaques, as their impairments in lipid metabolism and the immune system are otherwise relatively subtle (35, 36). A key advantage of this model is its biochemical similarity to human hypercholesterolemia, as the majority of cholesterol is carried by LDL particles, resulting in a lipid profile closer to that of humans (37). Because *Ldlr* knockout mice require dietary cholesterol to develop robust hypercholesterolemia and atherosclerosis, this model is particularly valuable for studying both the induction and prevention of the disease (38). However, the reliance on a Western diet to accelerate atherogenesis introduces variability in experimental results, making standardized cross-laboratory comparisons more challenging (39). Furthermore, since *Ldlr* knockout mice lack LDLR, they are not suitable for evaluating potential drugs that upregulate LDLR [such as proprotein convertase subtilisin/kexin type 9 (PCSK9) inhibitors] (40).

### Comparison of two mouse models

*Ldlr* knockout mice and *Apoe* knockout mice represent the two most extensively used atherogenic murine models, both relying on non-HDL hyperlipidemia to drive disease progression. While sharing mechanistic foundations, they exhibit key phenotypic and methodological distinctions: *Apoe* knockout mice display heightened systemic inflammation and accelerated plaque development even on standard chow, whereas *Ldlr* knockout mice exhibit milder immune perturbations and require cholesterol-enriched diets to induce robust hypercholesterolemia and atherosclerosis (26, 41). This dietary dependence of *Ldlr* knockout mice has prompted the use of double-knockout models to achieve rapid, diet-independent severe atherosclerosis (39, 42).

Metabolic Comorbidities: *Ldlr* knockout mice display heightened susceptibility to diet-induced type 2 diabetes (T2D) compared to *Apoe* knockout and wild-type controls, enabling studies of diabetes-atherosclerosis interplay. Additionally, *Ldlr* knockout mice show heightened susceptibility to diet-induced type 2 diabetes (T2D), enabling studies of diabetes-atherosclerosis interplay. Recent technical advancements, such as AAV-PCSK9 delivery, allow rapid induction of hyperlipidemia and plaque formation without prolonged dietary manipulation or germline *Ldlr* knockout (43, 44). Despite their utility, critical challenges persist, particularly variability in diet composition and induction protocols across laboratories, which compromises result reproducibility and cross-study comparability (41) (Table 1).

**Table 1:** Comparison of *Apoe* and *Ldlr* knockout mice models.

Feature	*Apoe* knockout mice	*Ldlr* knockout mice
Lipidprotein profile	VLDL predominant	LDL predominant (human-like)
Diet requirement	Standard chow sufficient	Western diet required
Lesion development	Spontaneous, rapid, severe	Diet-induced, diet-dependent
Systemic inflammation	High	Mild
T2D susceptibility	Low	High
LDLR-targeting drugs	Suitable (LDLR intact)	Not suitable (LDLR absent)
Best application	Spontaneous atherosclerosis, inflammation studies	Diet-induced atherosclerosis, graded disease models, diabetes interplay

## Adeno-associated virus-based models

Beyond these traditional germline knockout models, adeno-associated virus (AAV)-based approaches have emerged as a complementary and increasingly popular strategy in atherosclerosis research. AAV is a small, non-enveloped, single-stranded DNA virus and one of the most widely used gene delivery vectors in biomedical research (45). Its non-pathogenic nature in humans, combined with low immunogenicity and the capacity to sustain long-term transgene expression in non-dividing cells such as hepatocytes and endothelial cells, renders it particularly suitable for *in vivo* gene transfer applications (45). Furthermore, a variety of naturally occurring and engineered serotypes (including AAV8 and AAV9) exhibit distinct tissue tropisms, enabling targeted delivery to specific organs; for instance, AAV8 and AAV9 show high transduction efficiency in the liver, the central organ regulating systemic lipid metabolism and a key driver of atherosclerotic pathogenesis (26, 46). In atherosclerosis research, AAV vectors serve dual purposes: first, as tools for the rapid and cost-effective establishment of disease models in wild-type mice, bypassing the time-consuming breeding of conventional germline knockout strains; and second, as therapeutic gene delivery platforms for potential interventions, including the delivery of genes encoding lipid-lowering proteins, anti-inflammatory cytokines, or CRISPR-based gene-editing components aimed at permanent modification of pathogenic targets (47, 48). Despite persistent hurdles such as pre-existing neutralizing antibodies, dose-dependent hepatotoxicity, and limited packaging capacity, ongoing innovations in capsid engineering and tissue-specific promoters continue to expand the utility of AAV for both mechanistic studies and translational applications in atherosclerosis.

## Mouse models targeting different cells in atherosclerotic plaques

Both arterial wall cells and immune cells are involved in the development of atherosclerosis, including endothelial cells, smooth muscle cells, fibroblasts, neutrophils, mono-macrophages, T cells, B cells and dendritic cells (49). With the deep research into atherosclerosis development, recent studies have focused on the role of individual genes in specific cell types in regulating atherosclerosis development. Since global gene knockout mouse often trigger compensatory pathways that can mask direct mechanistic roles, cell-specific overexpression or knockout mouse models are frequently employed (4). Given the complex interactions among multiple cell types, selecting the optimal mouse model is both important and challenging for researchers. Here, we summarize the advantages and limitations of the most commonly used and recently developed mouse models in atherosclerosis, aiming to help researchers select the most appropriate model for their specific questions and to identify which models require further optimization.

### Mouse models targeting endothelial cells

Endothelial cells (ECs) play a critical role in maintaining vascular homeostasis, regulating vessel wall tension, inhibiting platelet aggregation and interacting with circulating blood components (50). Alterations in endothelial function lead to inflammatory cell infiltration, vascular smooth muscle proliferation, and platelet aggregation (51). Endothelial dysfunction is an early event in atherosclerotic lesion formation and a critical step in prompting inflammatory cells to adhere to blood vessel walls and trigger plaque formation, which finally leads to cardiovascular disease (52). The following section briefly outlines the advantages and limitations of the Cre mouse models most frequently used to target ECs in atherosclerosis (Table 2).

**Table 2:** Commonly employed EC-expressing Cre driver lines.

Mouse line	Advantages	Limitations	Ref.
*Tie2^Cre^, Tie2^CreER^*	Broad EC targeting,	Also expressed in bone marrow-derived cells; potential developmental adaptation	(54, 76–82),
*Cdh5^Cre^, Cdh5^CreER^*	Targets vascular ECs and allows tracking of EC function	Unintended activity in retinal ganglion cells, brain neurons, and myeloid cells	(56, 83–85)
*Flk1^Cre^*	labels diverse endothelial subtypes	Expression declines in adult quiescent endothelium	(62)
*Pdgfb^CreER^*	Targets ECs during development and adulthood	Weakly expression in pericytes and SMCs, potentially confounding EC-specific conclusions	(86)
*Lyve1^Cre^, Lyve1^CreER^*	Targeting lymphatic and liver sinusoidal ECs	Not expressed in arterial ECs (primary plaque site)	(87–89)
*Prox1^CreER^*	Targets lymphatic ECs	Also expressed in non-ECs (hepatocytes, neural progenitors, and intestinal cells)	(67, 68, 90, 91)

The *Tie2^Cre^* transgenic mice are the most commonly used for studying EC contributions to atherosclerosis but require careful experimental design to mitigate limitations (53). Key strengths include broad EC targeting and compatibility with disease models, while challenges include hematopoietic off-target effects and developmental confounding (54). Pairing Tie2-Cre with inducible systems and rigorous controls ensures robust conclusions about EC-specific mechanisms.

The *Cdh5^Cre^* (Cadherin 5 promoter-driven Cre recombinase) mouse model utilizes the Cdh5 promoter to drive endothelial-specific Cre recombinase expression (55), making it a valuable tool for studying atherosclerosis mechanisms, including endothelial dysfunction, inflammatory signaling, and vascular remodeling (56). Its primary advantage is precise endothelial lineage tracing (57), enabling investigations into cell turnover, plasticity, and shear stress responses during disease progression (58). Additionally, it facilitates detailed analysis of oxidative stress, nitric oxide signaling, and flow-dependent vascular adaptation (59). However, the model has limitations: altered leukocyte recruitment due to modified endothelial adhesion molecule expression may deviate from physiological conditions (60), and genetic modifications could disrupt endothelial repair mechanisms and lipid transport, potentially influencing cholesterol metabolism in the vessel wall. These factors must be carefully accounted for when interpreting experimental outcomes (61).

*Flk^Cre^* (Fetal Liver Kinase 1) transgenic mice are suitable for studying endothelial contributions to atherosclerosis from early developmental stages, as Flk1 is essential for vasculogenesis and angiogenesis (62).

*Pdgfb^Cre^* (platelet-derived growth factor subunit B) mice are another valuable mouse model for studying EC-specific pathways in atherosclerosis (63), particularly those involving shear stress and vascular development. However, their utility is constrained by developmental confounding, mosaic recombination, and off-target effects in pericytes/smooth muscle cells (SMCs) (64). Pairing with inducible systems, rigorous validation, and complementary models enhances their reliability for dissecting EC contributions to plaque biology.

*Lyve1^Cre^* (Lymphatic Vessel Endothelial Hyaluronan Receptor 1) mice are a specialized tool for studying lymphatic and liver sinusoidal endothelial cells in atherosclerosis (65, 66). Their strengths include specificity for Lymphatic Endothelial Cells (LECs)/Liver Sinusoidal Endothelial Cells (LSECs) and minimal myeloid off-target effects, but their utility is limited by poor relevance to arterial ECs and sparse expression in adult Blood Endothelial Cells (BECs). This model is best suited for niche studies on lymphatic-plaque crosstalk or hepatic lipid metabolism, requiring complementary arterial EC models (e.g., *Cdh5*^Cre^) for comprehensive insights.

*Prox1^Cre^* (Prospero homeobox protein 1) mice, driven by the Prox1 promoter (a lineage-defining marker for LECs), are ideal for studying lymphatic-specific mechanisms in atherosclerosis (67, 68). Unlike pan-endothelial Cre lines (e.g., *Tie2^Cre^*, *Cdh5^Cre^*), *Prox1^Cre^* avoids arterial BEC confounding and provides high LEC specificity with minimal EC interference, enabling exclusive analysis of lymphatic contributions (69). However, off-target non-EC expression and limited relevance to arterial plaques require complementary arterial-specific models such as *Cdh5^Cre^* for vascular studies.

*Bmx^CreERT2^* (Bone marrow tyrosine kinase on chromosome X) mice are a tamoxifen-inducible transgenic line that targets arterial ECs via the *Bmx* promoter, a Tec family kinase specifically expressed in arterial endothelium and endocardium (70, 71). Originally developed to investigate the role of Notch signaling in postnatal arteries (72), this model has recent been successfully employed this model to demonstrate that endothelial CD40L serves as an adhesion molecule in aortic, peritoneal, mesenteric, and coronary inflammation (73). Although not yet widely adopted in atherosclerosis research, its key advantages, including high arterial specificity with minimal venous EC recombination, efficient (>95%) inducible recombination, and postnatal compatibility that circumvents embryonic lethality, highlight its potential (72, 74). However, limitations include endocardial expression, expression in bone marrow epithelial cells and hematopoietic cells, late onset, mosaic recombination, and incomplete arteriolar coverage, necessitating validation with reporter lines (74, 75).

### Mouse models targeting smooth muscle cells

Vascular smooth muscle cells (VSMCs) make up the muscle layer of arteries and veins and are critical for the regulation of vascular tone and blood pressure (92). Under physiological conditions, VSMCs are differentiated and quiescent, but when injured, VSMCs undergo dedifferentiation, manifested by proliferation, migration, and downregulation of contractile protein. Dedifferentiation of VSMCs is central to vascular pathologies such as atherosclerosis, restenosis, and aneurysms (93).

SMC-associated Cre mouse models are indispensable tools in atherosclerosis research, enabling targeted genetic manipulation to investigate SMC contributions to disease pathogenesis (94). These models allow precise interrogation of SMC phenotypic transitions, lesion formation, and plaque progression, providing critical insights into atherosclerosis development and potential therapeutic interventions (95). By offering a genetically tractable platform, SMC-Cre models facilitate mechanistic studies of SMC-derived cells in vascular remodeling and plaque stability. Below we highlight key SMC-related Cre strains and their applications in atherosclerosis research (Table 3).

**Table 3:** Commonly employed SMC-expressing Cre driver lines.

Mouse line	Advantages	Limitations	Ref.
*Itga8^CreER^*	Targets arterial SMCs	Sex-specific effects	(110, 114, 115)
*SM22α^Cre^ (Tagln^Cre^)*	Expresses appropriately in SMCs	Expressed in myeloid cells, plateles, visceral SMCs, perivascular adipocytes and their precusors, and ECFs	(83, 94, 116–119)
*Myh11^CreER^, Myh11^CreNLS^*	Highly specific marker for mature SMCs	Expressed in pericytes, visceral SMCs, and low expressed in SMCs in advanced plaques	(110, 120–124)
*Acta2^Cre^, Acta2^CreER^*	Expressed in VSMCs across arteries, veins, and capillaries.	Expressed in myofibroblasts, pericytes, visceral SMCs, and activated stromal cells, and downregulation during phenotypic switching:	(94, 125)

*SM22α^Cr^*^e^ mouse model is widely used to investigate the role of VSMCs in atherosclerosis. In this model, Cre recombinase expression is driven by the SM22α promoter, enabling targeted genetic manipulation in SMCs to study their involvement in plaque formation and arterial wall thickening (96). Utilizing this model, researchers have revealed that AKT serine/threonine kinase 1 (Akt1) is specifically expressed in VSMCs and attenuates plaque progression through an incompletely defined mechanism (97). VSMC autophagy has also been shown to be critical in regulating the contractile and relaxation properties of small muscular arteries (98). Furthermore, SMC-specific Phosphatase and Tensin Homolog (PTEN) deficiency was found to promote vascular calcification, with Forkhead Box O1 and O3 (FOXO1/3) playing a key role in PTEN/AKT-mediated Runt-related Transcription Factor 2 (Runx2) ubiquitination and VSMC calcification (99). Another study defined the role of SMC-derived Runx2 in promoting vascular calcification and recruiting macrophages into calcified lesions to form osteoclast-like cells (100). Additionally, Neural precursor cell expressed developmentally down-regulated 4 (Nedd4) E3 ligase may contribute to VSMC calcification by activating osteogenic signaling during atherosclerosis progression (101). In lipid metabolism, SMC-specific knockdown of SREBP Cleavage-Activating Protein (*SCAP*) reduced lipid accumulation and oxidative stress, while enhancing lipid clearance via Reactive Oxygen Species (ROS)/AMP-Activated Protein Kinase (AMPK) pathway-mediated lipophagy, thereby alleviating atherosclerotic plaque formation (102). Moreover, VSMC-expressed Membrane Type 1 Matrix Metalloproteinase (MT1-MMP) limits atherosclerosis progression in *Apoe* knockout mice by modulating proliferative responses and preserving VSMC function in atherogenic vascular walls (103). Collectively, these findings highlight the diverse roles of VSMC-specific genes in regulating plaque formation, calcification, lipid metabolism, and vascular remodeling.

*Myh11^CreERT2^* mouse has revolutionized the study of SMC biology in atherosclerosis by enabling inducible lineage tracing and gene manipulation in mature SMCs. This capability has been instrumental in revealing the remarkable phenotypic plasticity of SMCs, demonstrating their ability to contribute to atherosclerotic plaque pathogenesis by downregulating classic markers like Myh11 and adopting macrophage-like or other non-canonical fates, a process critically dependent on factors such as Krüppel-like Factor (KLF) (104). However, this plasticity creates ambiguity in definitively distinguishing SMC-derived cells from genuine leukocytes or other lineages in advanced plaques. Furthermore, a technical limitation of the widely used first-generation model is its Y-chromosome linkage, which restricts its utility to male mice (105); this has since been circumvented by the development of sex-independent models, including the Owens group's *Myh11^CreERT2−RAD^* strain and other recently reported lines which enable SMC-specific targeting in both male and female mice (106–108).

Integrin subunit alpha 8 (Itga8) is a subunit of the α8β1 integrin receptor. During embryonic development, it is predominantly expressed in VSMCs, with only minimal expression in visceral SMCs, and this expression pattern is maintained in mature contractile SMCs (109). Thus, unlike traditional SMC-Cre models mentioned above, the *Itga8^CreERT2^* model offers unparalleled precision for vascular SMC research in atherosclerosis, circumventing visceral SMC confounding effects (110). However, studies in *Apoe* and *Itga8* double konckout mice global knockout mice have revealed sex differences in atherosclerosis severity and renal injury, suggesting that Itga8 may interact with sex hormones or immune pathways; whether this applies to Itga8^Cre^-mediated conditional deletion requires further investigation (111). More importantly, the activity of *Itga8^Cre^* may be reduced in advanced plaques due to the downregulation of Itga8 in phenotypically modulated SMCs (112, 113).

### Mouse models targeting monocytes and macrophage

In atherosclerosis, peripheral blood monocytes are continuously recruited to plaques, with their numbers increasing in proportion to plaque size. Macrophages are the main immune cells responsible for plaque formation, development, and rupture, and they become particularly abundant in advanced lesions (126). Below, we summarize the commonly used Cre mouse models targeting monocytes and macrophages (Table 4).

**Table 4:** Commonly employed Mon-macrophage expressing Cre driver lines.

Mouse line	Advantages	Limitations	Ref.
*LysM^Cre^, LysM^CreER^*	Targets macrophages and neutrophils	Not macrophage specificity; Neutrophil off-target effects	(165–168)
*CX3CR1^CreER^*	Targets multiple macrophages and classical dendritic cells	off-target effects, partial myeloid coverage, and variable Cre efficiency.	(169, 170)
*CD11b^Cre^*	Myeloid targeting, early monocyte recombination.	Off-target effects, variability, poor specificity	(171)
*CD68^CreER^*	Macrophage-specific, temporal control	CD68 variability, inconsistent recombination, limited early monocyte targeting.	(172)
*F4/80^Cre^*	Targets tissue-resident macrophages	Monocyte limitation, variable activity, incomplete deletion, off-target.	(173)
*Mafb^Cre^*	Distinguish macrophages from other immune lineages	Also expressed outside the hematopoietic system	(174)

*LysM^Cre^* (lysozyme M, encoded by Lyz2) and *LysM^CreER^* mouse models are widely used in atherosclerosis research to study foam cell formation (127), inflammatory responses (128) and macrophage-driven plaque progression (129), as *LysM^Cre^* exhibited strong recombinase activity in macrophages (130). However, LysM expression is stronger in circulating monocytes, limiting its effectiveness for plaque-resident macrophages (131), and is also expressed in neutrophils, causing off-target recombination and complicating the isolation of macrophage-specific gene effects in atherosclerosis (132, 133).

The CX3C Chemokine Receptor 1 (CX3CR1) chemokine receptor is expressed on proatherogenic myeloid cells, including inflammatory monocytes, macrophages and dendritic cells (134, 135). Therefore, *CX3CR1^Cre^* mice are widely used to study the role of monocytes, macrophages, and dendritic cells (DCs) in atherosclerosis (136). As CX3CR1^+^ monocytes interact with endothelial cells during adhesion and transmigration, CX3CR1Cre models help dissect this interaction in early atherogenesis (137). However, CX3CR1 is also expressed on non-classical monocytes and microglia, confounding atherosclerosis specific conclusions (138, 139). Additionally, it should be noted that classical Ly-6C^high^ monocytes express low levels of CX3CR1 and are the dominant subset recruited during early atherogenesis (140).

CD11b is another broadly expressed marker in myeloid cells, including monocytes, macrophages, some dendritic cells and some natural killer cells (NK cells). *CD11b^cre^* mice are particularly useful for studying monocyte-derived macrophages in atherosclerotic plaques and their contributions to disease progression (141). CD11b is expressed early in monocyte differentiation, enabling gene recombination during the initial stages of monocyte-to-macrophage transition. Its expression supports research on monocyte recruitment and their differentiation into foam cells in atherosclerosis (142), and macrophage polarization between pro-inflammatory (M1) and anti-inflammatory (M2) states (143). However, its utility is limited in studying resident macrophage populations within atherosclerotic plaques, as CD11b expression is not restricted to plaque macrophages but is also present on circulating monocytes and other myeloid cells (144).

The *CD68^CreER^* mouse model is widely used to study macrophages-specific contributions to inflammation (145), lipid uptake (146), and plaque stability in atherosclerosis (147). It enables fate tracking of resident and recruited macrophages in plaques, distinguishing their respective roles in disease progression (148). Tamoxifen induction allows time-controlled gene recombination, facilitating analysis of macrophage functions at different disease stages (149). However, CD68 is primarily expressed in differentiated macrophages, limiting its utility for studying early monocyte recruitment or progenitor cells (150). Additionally, CD68 expression in other myeloid cells may lead to non-macrophage Cre activity (151).

F4/80 is highly expressed in tissue-resident macrophages but weakly in monocytes and neutrophils (152–154). Thus, *F4/80^Cre^* mice enable targeted gene recombination primarily in resident macrophages, making them valuable for dissecting their roles in atherosclerosis progression (155). Monocyte-derived macrophages in atherosclerotic lesions can also express F4/80, making this model useful for studying polarization (156), foam cell formation (157), and inflammation in advanced plaques (158). However, this makes it difficult to distinguish their contributions from those of tissue-resident macrophages in advanced lesions (159). Furthermore, weak F4/80 expression in circulating monocytes makes it less effective for studying early monocyte recruitment and differentiation (160). Expression in dendritic cells and eosinophils may lead to unintended recombination in non-macrophage immune cells (159).

CSF1R (macrophage colony-stimulating factor receptor, or CD115) is a tyrosine kinase receptor encoded by c-fms and serves as the receptor for CSF1, the major differentiation factor for the monocyte/macrophage lineage. *Csf1r^Cre^* mice have been generated for tracing endogenous macrophages across various tissues, including the liver, spleen, intestine, heart, kidney, and muscle (161). Flow cytometry confirmed approximately 80% recombination in bone marrow-derived macrophages, but also revealed substantial off-target activity in approximately 50% of bone marrow granulocytes and splenic T lymphocytes (162, 163). Therefore, while Csf1r-Cre serves as a valuable tool for macrophage fate mapping and identification of macrophage regulatory genes essential for both steady-state and inflammatory conditions (164), its limited specificity, with additional activity in dendritic cells, granulocytes, and T cells (163), necessitates careful validation with reporter lines.

Most current systems for conditional gene deletion in mono-macrophage are limited by ectopic or low efficiency recombination. Future specific markers of intra-plaque inflammatory macrophages need to be discovered.

### Mouse models targeting neutrophils

Much of the neutrophil proinflammatory activity can be attributed to the release of granule proteins. Neutrophils also contribute to advanced atherosclerosis by secreting and activating various matrix metalloproteinases (e.g., MMP8) and cathepsins, which degrade the basement membrane and extracellular matrix components, thereby promoting plaque fragility, erosion, and rupture (175).

Several mouse models have been developed to study neutrophil function in atherosclerosis. The LysM^Cre^ Rosa26^loxP−STOP−loxP−DTR^ model enables expression of toxin receptors in LysM^+^ myeloid cells, including neutrophils, monocytes, and macrophages, allowing depletion of these cells upon diphtheria toxin (DT) administration (176). However, because this model targets multiple myeloid lineages, it is not specific to neutrophils. To achieve neutrophil-selective depletion, the *LY6G^DTR^* mouse model has been developed, in which DTR is specifically expressed on LY6G⁺ neutrophils, enabling selective neutrophil elimination after DT injection (177). This model is used to assess whether neutrophil depletion slows or reverses atherosclerosis progression, providing an experimental basis for neutrophil-targeted therapies (178).

*S100a8^Cre^* mice are a transgenic line that utilizes the S100A8 (MRP8) promoter to drive Cre recombinase expression for neutrophil-specific gene targeting in atherosclerosis research (179). For instance, S100A8-Cre-mediated ABCA1/G1 double knockout promotes atherosclerosis through increased cholesterol accumulation and enhanced NETosis in neutrophils (180). Although S100A8-Cre mediates efficient recombination in mature neutrophils, its specificity is incomplete due to low-level expression in other myeloid cells such as monocytes (170, 181). Additionally, random transgene integration may cause off-target genomic deletions, such as Serpine1 and Ap1s1 (182).

### Mouse models targeting dendritic cells

Dendritic cells (DCs), whether in lymphoid or nonlymphoid tissues, comprise only a minor portion of the total cell population, and are also scarce in human and mouse plaques, but they play a unique role in the development and progression of atherosclerosis. They influence atherosclerosis by production of chemokines and cytokines, antigen presentation, and lipid uptake either promoting inflammation or inducing tolerance (183). Below, we summarize commonly used DC-related Cre strains and their applications in atherosclerosis research (Table 5).

**Table 5:** Commonly employed DC-expressing Cre driver lines.

Mouse line	Advantages	Limitations	Ref.
*CD11c^Cre^*	Enables DC-specific gene editing to study their role in atherosclerosis with good comparability	Incomplete representation of DC heterogeneity, challenges in studying DC migration,	(184, 187, 188)
*Zbtb46^Cre^*	Targets specific genes in Zbtb46^+^ DCs and distinguishes cDCs from other lymphoid and myeloid cells	Zbtb46 downregulated after DC stimulation and is additionally found on ECs, early erythroid progenitors, and monocytes.	(193, 194)
*Clec9a^Cre^*	Marks cells traditionally ascribed to the DC lineage, and this restriction is maintained after inflammation	Ambiguity for pDCs	(200)
*Xcr1^Cre^*	Direct assessment of the necessity of cDC1s in disease progression	–	(201)

The *CD11c^Cre^* (Itgax) mouse model, engineered with Cre recombinase inserted under the control of CD11c promoter, has become an essential tool for investigating DC biology in atherosclerosis. CD11c expression is predominant in conventional DCs (cDCs) enables targeted genetic manipulation to study DC-specific functions (184). This well-characterized model has significantly advanced our understanding of DC-mediated immune regulation, including effects on T helper 1 (Th1)/Th2 polarization and regulatory T cell (Treg) induction, and plaque inflammatory dynamics (185). Its extensive use in immunology and atherosclerosis research provides a robust framework for cross-study comparisons (186). However, limitations include off-target recombination in other CD11c⁺ myeloid populations, incomplete recombination efficiency that fails to capture the full DC spectrum in plaques, and an inability to distinguish resident from migratory DC subsets, all of which stem from ubiquitous CD11c expression (187, 188). Additionally, compensatory immune adaptations following DC-specific perturbations may obscure mechanistic interpretations (189).

The *Zbtb46^cre^* (Zinc Finger and BTB Domain Containing 46) mouse model provides a targeted approach to investigate cDC functions in atherosclerosis, thereby allowing researchers to dissect their specific roles in plaque-associated immunoinflammatory processes (190). A key advantage of this model is its ability to distinguish cDCs from other lymphoid and myeloid cells. Thus, it can be used for precise identification of Zbtb46^+^ DC contributions to disease pathogenesis, elucidation of DC-mediated immune regulation (including T-cell modulation, cytokine networks, and metabolic pathways), and characterization of DC-dependent monocyte-macrophage interactions (190–192). However, the expression of Zbtb46 is downregulated after DC stimulation and is additionally found on endothelial cells, early erythroid progenitors, and monocytes stimulated with Granulocyte-Macrophage Colony-Stimulating Factor (GM-CSF) and IL-4 (193, 194).

The *Xcr1^Cre^* mouse targets the XCR1⁺ subset of conventional type 1 dendritic cells (cDC1s) and is widely used to study cDC1 lineage commitment, function, and immune responses (195–198). While earlier versions exhibited documented Cre leakiness in other immune cells (195), an optimized *Xcr1^Cre^* model shows no detectable leakage. Notably, crossing this improved model with Apoe knockout mice expressing Diphtheria Toxin A (DTA) definitively established that cDC1s promote atherosclerosis (199).

### Mouse models targeting T cells

Converging evidence from preclinical mouse models, clinical interventions, and advanced molecular techniques underscores T cells as pivotal drivers and modifiers of atherosclerosis pathogenesis (202). CD4^+^ T cells, also known as helper T cells, are abundant in atherosclerotic plaques. They regulate immune responses in atherosclerosis, participate in inflammation mediation, and promote plaque formation and stability (203). CD8^+^ T cells, also called cytotoxic T cells, are mainly responsible for identifying and killing infected or damaged cells, thereby playing a role in immune surveillance and clearing damaged cells in atherosclerosis (204). Th1 cells typically worsen plaque development, while Treg cells usually protect against it, though Tregs can sometimes become harmful. The roles of other T cell types, including Th2, Th17, and γδ T cells) are less clear, and some T cells may have mixed effects. This section summarizes studies using mouse models to explore how T cells migrate to plaques, including their benefits and limitations (Table 6).

**Table 6:** Commonly employed T cell expressing Cre driver lines.

Mouse line	Advantages	Limitations	Ref.
*CD4^Cre^*	T cell targeting CD4⁺ function	Early recombination T cell development	(206, 207)
*CD8^Cre^*	CD8⁺ T cell targeting, immune memory.	T cell recombination, subset resolution,	(208, 209, 212)
*Lck^Cre^*	T cell targeting, adaptive immunity, subset analysis.	limited control, thymic development, T cell differentiation.	(215, 222, 223)
*Foxp3^Cre^*	Treg-specific targeting, immune tolerance	Treg development, functional plasticity, expression variability	(224, 225)
*RORγt^Cre^*	Th17/*γδ* T cell targeting, cytokine signaling	functional plasticity, data interpretation challenges	(230, 233)
*T-bet^Cre^*	Th1 regulation, FN-γ signaling, plaque stability, macrophage activation	T cell differentiation, inflammatory variability, Th1 restriction.	(235, 236)

*CD4^Cre^* mice are widely used for T-cell-specific gene knockout (205), and serve as a key tool to study the role of T cells in atherogenesis (206). By using *CD4^Cre^* mice, researchers can dissect the functions of different T cell subsets (such as Th1, Th2, Th17 and Treg) in atherosclerosis, providing both theoretical and experimental basis for gene therapy (207).

The *CD8^Cre^* mouse model is widely used to study T cell-mediated inflammation, including CD8⁺ T cell cytotoxicity, cytokine secretion [interferon-gamma (IFN-γ), Tumor Necrosis Factor-alpha (TNF-α)], antigen-specific memory CD8⁺ T cells, and immune activation in plaques (208, 209). This model also help assess how CD8⁺ T cells influence smooth muscle cells, endothelial function, and macrophage activation (210). However, Early Cre activity in thymic selection may alter T cell differentiation and immune balance (211), and it cannot distinguish between naïve, effector, and memory CD8⁺ T cells, complicating analysis (212). Furthermore, CD8 expression in thymic precursors may lead to gene recombination in CD4⁺ T cells, affecting lineage specificity (213), and low CD8 expression in dendritic cells and innate lymphoid cells may cause unintended recombination (214).

*Lck^Cre^* (lymphocyte-specific protein tyrosine kinase) mice are widely used to study T cell activation (215), cytokine secretion [IFN-γ, IL-2 and Transforming Growth Factor-beta (TGF-β)] (216–218), and regulatory functions in plaque progression (219). as Lck is a key kinase in T cell receptor (TCR) signaling expressed during T cell development in the thymus and in mature peripheral T cells. This model allows the examination of CD4⁺ helper T cells (220), CD8⁺ cytotoxic T cells (221), and Tregs in vascular inflammation (222). On the other hand, Cre activity begins in double-negative thymocytes, potentially influencing T cell selection, differentiation, and immune homeostasis before atherosclerosis onset (223).

*Foxp3^cre^* mice enable Treg-specific genetic modifications, making it a valuable tool for studying targeted gene in Foxp3⁺ regulatory T cells in immune tolerance and vascular inflammation (224, 225). Furthermore, this model helps assess how Tregs regulate smooth muscle cell function, extracellular matrix remodeling, and local immune responses, influencing plaque composition (226), and enables study of the role of Treg-derived cytokines (IL-10, TGF-β) and immune checkpoint molecules in atherosclerotic lesion development (227). However, Foxp3 expression fluctuates under inflammatory conditions, which may lead to heterogeneous Cre activity across different disease stages (228). Additionally, due to the phenotypic and functional plasticity of Foxp3⁺ Tregs, the *Foxp3^Cre^* mouse model may not fully capture their conversion into pro-inflammatory subsets in disease settings (229).

The *RORγt^cre^* (Retinoid-related Orphan Receptor gamma t) mouse model is useful for studying the contribution of Th17 and γδ T cells in endothelial dysfunction (230), macrophage activation (231) and leukocyte recruitment (232) in atherosclerosis. However, it is challenging to define the precise contributions of targets genes at different stages of atherosclerosis, as RORγt⁺ T cells display context-dependent plasticity (233). Additionally, the involvement of RORγt⁺ T cells in both protective and pathogenic immune responses may complicate the interpretation of their role in atherosclerosis (234).

Th1 cells play an important role in the development of atherosclerosis by secreting cytokines such as IFN-γ to promote inflammatory responses (235). T-bet (also known as Tbx21) is a transcription factor critical for Th1 cells differentiation, IFN-γ production, and immune regulation. The *T-bet^Cre^* mouse model provides a powerful tool for studying the role of target genes in Th1 cells in atherosclerosis (235, 236). However, T-bet expression fluctuates in response to cytokine signals and disease conditions, which may lead to heterogeneous Cre activity and differential gene modifications (237). T-bet is also expressed in NK cells, CD8^+^ T cells, and subsets of Tregs, which may exert opposing effects on plaque development, further complicating data interpretation.

### Mouse models targeting B cells

Although only a small number of B cells reside within atherosclerotic plaques, with the majority localized in the adventitia, they exert important regulatory functions in the development of atherosclerosis (238). Beyond this local presence, systemic studies have revealed a more complex functional landscape among B cell subsets (239). B1 cells, a minor fetal-derived population that generates natural immunoglobulin M antibodies, are widely considered athero-protective (240, 241). Marginal zone B cells represent a non-circulating splenic subset that mediates an innate-like, rapid antibody response to blood-borne antigens and likewise exhibits athero-protective capabilities (242). Follicular B cells are essential for durable T cell-dependent antibody responses and have been reported as both pro- and anti-atherogenic (243–245).

To date, most studies on B cells in atherosclerosis have relied on the μMT (μ heavy chain membrane exon targeted) mouse model, which completely ablates all B cells to assess their net contribution (11). In contrast, B cell-specific Cre mice have rarely been used in atherosclerosis research. Among the available tools, *Cd19^Cre^* is the most commonly used (246). However, it carries a critical caveat: insertion of the Cre cassette into the endogenous Cd19 locus leads to *Cd19* haploinsufficiency, which alone alters B-cell homeostasis and, notably, attenuates atherosclerotic lesion development in hypercholesterolemic mice (247, 248). Consequently, when the *Cd19^Cre^* system is employed in atherosclerosis studies, *Cd19*^cre/+^ littermates, which exhibit the same *Cd19* haploinsufficiency—must be used as controls to distinguish Cre-mediated effects from those attributable to *Cd19* deficiency alone.

With the evolving understanding of B-cell subset biology, additional B-cell-specific Cre lines have been developed to enable more refined genetic manipulations, including *Mb1^Cre^* (*Cd79a^Cre^*), *AID^Cre^* (*Aicda^Cre^*), *Cd21^Cre^*, and *IgJ^Cre^* (249–252). Their current applications in atherosclerosis research, along with their respective advantages and limitations, are summarized in Table 7.

**Table 7:** Commonly employed B cell expressing Cre driver lines.

Mouse lines	Advantages	Limitations	Ref.
*Cd19^Cre^*	Most widely used (from pro-B stage)	*Cd19* haploinsufficiency alters B-cell homeostasis and lesion development	(246–248)
*Mb1^Cre^ (Cd79a^Cre^)*	High specificity and early B-cell targeting	Lower Cre activity in early B cells compared to *CD19^iCre^*; may affect pre-BCR signaling	(250)
*AID^Cre^ (Aicda^Cre^)*	Targets antigen-experienced B cells (GC, memory, and plasma)	Disrupts central tolerance, boosts autoantibodies and reduces autoreactive B cell apoptosis.	(251)
*Cd21^Cre^*	Targets mature B cells (marginal zone and follicular B cells)	Expression is restricted to mature stages, thus unsuitable for early B-cell development studies	(249)
*IgJ^Cre^*	Highly specific for plasma cells	Limited to terminal plasma cell stage, cannot study earlier B-cell differentiation or function	(252)

### Mouse models targeting fibroblast

Fibroblasts play a crucial role in the pathogenesis of atherosclerosis by regulating extracellular matrix production, contributing to plaque stability, and participating in vascular remodeling (253). To explore these functions *in vivo*, several fibroblast-specific mouse models have been developed and widely used in atherosclerosis research (Table 8).

**Table 8:** Commonly employed fibroblast expressing Cre driver lines.

Mouse line	Advantages	Limitations	Ref.
*Col1a1^CreER^*	Fibrosis investigation, Collagen regulation insights, Structural plaque research	Anatomical inconsistency, Transcriptional fluctuation, Early-phase limitations	(254, 257)
*Tcf21-MerCreMer*	Effectively tracks fibroblast roles in plaque progression and ECM remodeling	Incomplete fibroblast targeting, favors adventitial cells, and has lineage tracing limitations	(259, 260)
*PDGFRα^CreER^*	Enables tamoxifen-inducible, broad fibroblast labeling for studying vascular remodeling in atherosclerosis	Limited by variable recombination efficiency, fluctuating PDGFRα expression, and regional vascular bias.	(260–262)
*Postn-Cre*	Targets activated fibroblasts for studying plaque stability and ECM remodeling in advanced atherosclerosis.	Limited to late-stage disease, lacks healthy tissue expression, and misses quiescent fibroblast populations.	(264–266)

The tamoxifen-inducible *Col1a1^CreER^* (Collagen Type I Alpha 1 Chain) mouse model targets collagen-producing fibroblasts, enabling temporally controlled investigation of their roles in ECM remodeling and plaque stability in atherosclerosis (254, 255). As a well-validated tool, it provides reproducible insights into collagen deposition, fibrous cap formation, and plaque architecture in vascular disease (256). However, its utility is constrained by regional variability in recombination efficiency, dynamic *Col1a1* expression that depends on disease stage and the local microenvironment, and limited applicability for studying early fibroblast activation, as *Col1a1* expression is predominant during advanced fibrosis (257).

The *Tcf21^MerCreMer^* (Transcription factor 21) mouse model utilizes a tamoxifen-inducible Cre recombinase driven by the Tcf21 promoter to enable temporal gene manipulation in mesenchymal-derived fibroblasts during atherosclerotic lesion development (258). This model is particularly valuable for tracking fibroblast contributions to plaque progression (259), investigating fibroblast-to-smooth muscle cell transitions, and analyzing their role in ECM remodeling (260). However, its utility is limited by incomplete fibroblast coverage (missing low/transient Tcf21-expressing subpopulations), a bias toward adventitial fibroblasts over other vascular layers, and potential gaps in lineage tracing due to phenotypic plasticity of fibroblast-derived cell.

The *Pdgfrα^CreER^* (Platelet-Derived Growth Factor Receptor Alpha) mouse model, driven by a tamoxifen-inducible Cre under the Pdgfrα promoter, enables precise genetic modification of Pdgfrα-expressing fibroblasts to study their role in vascular remodeling during atherosclerosis (260). Its broad fibroblast labeling facilitates tracking of fibroblast behavior in plaque growth, stromal interactions, and extracellular matrix dynamics (261). However, limitations include variable recombination efficiency due to heterogeneous Pdgfrα expression, temporal fluctuations in Pdgfrα levels during disease progression, and regional bias from uneven Pdgfrα distribution across vascular tissues, all of which may skew fibroblast representation in analyses (262).

The *Postn^Cr^*^e^ mouse model, driven by the periostin promoter, specifically targets activated fibroblasts involved in atherosclerotic plaque development (263). It provides unique advantages for studying plaque stability by revealing how remodeling fibroblasts influence ECM deposition and plaque architecture, and enables detailed analysis of fibroblast-ECM interactions during disease progression (264). However, its utility is limited by late-stage specificity, as periostin expression primarily occurs during advanced remodeling, missing early pathogenic events (265). Additionally, low periostin expression in healthy tissue restricts normal-vs.-diseased comparisons its predominant labeling of activated fibroblasts may not fully represent quiescent fibroblast populations in plaques (266).

### Mouse models targeting NKT cells

Studies have found that NK cells exist in atherosclerosis lesions and may participate in the development of atherosclerosis by directly killing abnormal cells and producing cytokines (267, 268). However, because NKT cells are scarce in plaques (accounting for only 0.5%–5% of T cells), the need for NKT cell-specific Cre tools in atherosclerosis research has not been widely recognized. Most researchers have instead relied on global NKT-deficient mice or antibody blocking strategies.

A recent study using a novel *Ncr1^Cre^* model suggested absence or excessive activation of NK cells does not significantly affect atherosclerosis development, whereas activated NK cells may be pro-atherogenic (269). This *Ncr1^Cre^* model selectively eliminates NKp46⁺ cells (including NK cells, ILC1s, and a subset of ILC3) with near-complete efficiency and minimal off-target effects on T cells, B cells or myeloid cells, enabling reliable NK cell depletion in atherosclerosis studies. However, it lacks temporal control and cannot distinguish NK cells from ILC1/ILC3.

The *Zbtb16^Cre^Rosa26^DTR^* mouse represents a novel conditional model for NKT cell depletion. It enables depletion of NKT cells via DT without affecting conventional T cells, B cells or myeloid cells, although it also ablates other PLZF⁺ innate-like T cells such as mucosal-associated invariant T (MAIT) and γδ T cells (270). This model may be useful for future studies of NKT cells in atherosclerosis.

## Mouse models targeting platelet

Platelets participate in all stages of atherosclerosis, from early endothelial dysfunction to advanced plaque instability. Prior to the appearance of overt endothelial lesions at sites predisposed to atherosclerosis, platelets can deposit onto the vascular wall, induce endothelial dysfunction, and subsequently promote leukocyte adhesion to the endothelium (271).

The *Pf4^Cre^* (platelet factor 4) mouse is the most widely used platelet-specific Cre model, enabling highly efficient gene recombination in platelets (272). However, this model has a key limitation: Pf4 expression is not strictly restricted to the megakaryocytic lineage, and low-level ectopic expression can be observed in other myeloid cells, particularly under inflammatory conditions.

The *Gp1ba^Cre^* (glycoprotein Ibα) mouse exhibits higher specificity with reduced ectopic expression, albeit with lower recombination efficiency compared to *Pf4^Cre^* (273). Notably, a recent comparative study demonstrated that *Pf4^Cre^* and *Gp1ba^Cre^* -mediated deletion of cyclooxygenase-1 in platelets produced opposing effects on atherogenesis in *Ldlr* knockout mice, suggesting that the choice of Cre line can profoundly influence experimental outcomes and thus requires careful validation (274).

The tamoxifen-inducible *Itga2b^Cre^* (integrin α IIb) model enables temporally controlled gene deletion in platelets, which represents its main advantage. However, the Itga2b promoter also exhibits activity in early hematopoietic progenitors, embryonic hemogenic endothelium, and CD41⁺ tissue-resident myeloid cells in certain tissues. Consequently, upon tamoxifen induction, Cre recombination occurs not only in the megakaryocyte-platelet lineage but also in a subset of hematopoietic precursor cells (275).

## Mouse models targeting lipid metabolism

Dysregulated lipid metabolism serves as a critical driver throughout atherosclerosis development (276). Consequently, Cre mouse models targeting lipid metabolism-related tissues are essential for dissecting the mechanistic links between lipid homeostasis and plaque pathogenesis.

Three major organs govern systemic lipid metabolism: the liver, which is the primary site for lipoprotein synthesis and cholesterol clearance; the intestine, which modulates dietary lipid absorption; and adipose tissue, which serves as the major reservoir for lipid storage and release. Accordingly, researchers increasingly leverage hepatic-, intestinal-, and adipose-specific Cre models to investigate the mechanism of lipid metabolism in atherosclerosis. Commonly used models include hepatic lines such as *Alb^Cre^* (Albumin) and Tbg*^Cre^* (Thyroxine Binding Globulin) to study LDLR/PCSK9-mediated cholesterol clearance (277, 278), intestinal models such as *Villin^Cre^* to examine cholesterol absorption (279), and adipose models such as *Adipoq^cre^* (Adiponectin) to explore the crosstalk between lipolysis and inflammation (280).

## Mouse models targeting hematopoietic cells

Hematopoietic cells are central to atherosclerosis, as all plaque-infiltrating immune cells derive from bone marrow hematopoietic stem cells (281, 282). Cardiovascular risk factors drive hematopoietic stem cells expansion and myeloid-biased differentiation, boosting production of pro-inflammatory monocytes and neutrophils that infiltrate the vessel wall and contribute to plaque initiation, progression and destabilization. Atherosclerosis in turn further perturbs hematopoiesis, creating a feed-forward loop (283). Therefore, precise genetic targeting of hematopoietic cells is essential for dissecting the immune mechanisms of this disease.

*Mx1^Cre^* is an interferon-inducible transgenic line that enables temporally controlled gene deletion in hematopoietic cells upon pIpC injection (284, 285). It has been successfully applied in atherosclerosis research, revealing that Jak2V617F mutation accelerates plaque progression, Glut1 deletion suppresses myelopoiesis, and Plpp3 knockout increases plaque burden (286–288). However, its utility is constrained by off-target activity in non-hematopoietic tissues such as the liver and heart (289), along with transient pIpC-induced effects on HSPC phenotype and blood counts that may confound short-term studies (285). Rigorous controls, including Cre-negative and pIpC-treated controls, are therefore essential.

## The AAV8-PCSK9 model

The AAV8-PCSK9 model represents a novel approach for rapidly inducing atherosclerosis. This model involves a single tail-vein injection of a recombinant AAV8 encoding a gain-of-function mutant of proprotein convertase subtilisin/kexin type 9 (PCSK9), which targets hepatic LDLR for degradation. When combined with a high-fat diet, this leads to a rapid increase in circulating cholesterol levels and the development of atherosclerotic plaques (47, 290).

Unlike *Apoe* and *Ldlr* germline knockout mice, which require prolonged cross-breeding, the AAV8-PCSK9 method only requires a single tail-vein injection, thereby substantially shortening experimental timelines and reducing costs (291). Moreover, the dyslipidemia in *Apoe* knockout mice is characterized primarily by elevated VLDL, which differs substantially from the human pathological feature of elevated LDL-C. In contrast, the AAV-PCSK9 model primarily elevates plasma LDL cholesterol, making it more closely resemble human LDL-dominant atherosclerosis. This model is therefore particularly suitable for evaluating the efficacy of PCSK9 inhibitors, statins, and other LDLR pathway-targeting drugs (291). However, the AAV8-PCSK9 model also has limitations. While *Apoe* knockout mice develop plaques on a normal chow diet, AAV-PCSK9 mice exhibit only mild hyperlipidemia on standard chow and require a cholesterol-containing Western-type diet to induce significant atherosclerotic lesions. Notably, batch-to-batch and cholesterol-content variations in the diet can introduce inter-group variability, compromise experimental reproducibility and complicate direct cross-laboratory comparisons (292).

## Discussion

Animal models are indispensable for advancing our understanding of atherosclerosis pathogenesis and therapeutic development (293, 294). They facilitate the discovery of novel biomarkers and functional pathways (295), as well as target validation and drug screening using both phenotype-based and target-based approaches (296). To date, over 15,000 human disease-associated mutations spanning oncology, metabolism, neurology, and cardiology have been modeled in mice, offering unparalleled insights into mutation-driven pathologies and accelerating therapies for rare genetic diseases (297). These models are pivotal for pharmacokinetic profiling, therapeutic efficacy testing, and de-risking drug candidates prior to clinical translation (298).

Technological innovations continue to enhance model utility. Humanized mouse models, generated via xenotransplantation of human cells or tissues, bridge species-specific gaps by recapitulating human disease states. For example, the severely immunodeficient NOD/Shi-scid-IL-2Rγnull (NOG) mouse lacks functional T cells, B cells, and NK cells (299), making it an ideal host for engraftment with human immune cells to study their specific functions in atherosclerosis. Meanwhile, CRISPR/Cas9-mediated genome engineering enables precise pathogenic allele recapitulation, immune system modeling, and functional genomics studies (300, 301). As an illustration, AAV-delivered CRISPR/Cas9 systems have corrected *Ldlr* mutations in mouse models of familial hypercholesterolemia, demonstrating therapeutic potential for atherosclerosis-linked dyslipidemias (302). Such innovations not only advance mechanistic research but also refine preclinical drug development pipelines and improve clinical predictability (303).

Nevertheless, persistent limitations require acknowledgment (Table 9). Human atherosclerosis is a multifactorial disease shaped by genetic, environmental, and lifestyle interactions, whereas murine models often oversimplify pathology by focusing on single pathways like hyperlipidemia (304). Key physiological divergences, such as HDL-dominant lipid profiles, lack of cholesteryl ester transfer protein (CETP), and plaque localization to the aortic root rather than coronary arteries, and these differences limit direct extrapolation to human disease (305–307).

**Table 9:** Limitations of mouse models in atherosclerosis research.

Category	Key Limitations	Consequences
Species divergence	HDL-dominant lipid profiles CETP deficiency Aortic root (vs. coronary) plaque localization	Reduced clinical translatability
Pathophysiological gaps	Compressed disease timeline Strain-specific susceptibility Sex-dependent phenotypes	Limited human relevance
Technical constraints	Embryonic lethality of critical targets Artificial high-fat diets Surgical accessibility	Mechanistic oversimplification

Mice from different strains of mice may respond differently to experimental treatments or may affect atherosclerosis development, leading to variability in results. Therefore, focusing on the pathogenic pathways rather than individual genes is likely to be a more productive way to translate information from mouse models to human atherosclerosis (13). Additionally, rapid atherosclerosis progression in mice (weeks vs. decades in humans), strain-specific susceptibility (e.g., C57BL/6J), and sex-dependent phenotypes (female mice vs. male humans) introduce variability and reduce clinical relevance (308–310). Technical limitations, including small size complicating surgical interventions and reliance on artificial high-fat diets, further obscure mechanistic insights and therapeutic predictions (311).

The expanding identification of disease-relevant cellular subpopulations has driven growing demand for Cre-loxP technology, which is indispensable for studying tissue-specific mechanisms and embryonic lethal genes (312, 313). Therefore, systematically evaluating the advantages and limitations of diverse Cre-loxP models is essential to guide optimal model selection in atherosclerosis research. Although powerful for cell-specific genetic manipulation, these models are hampered by off-target recombination and developmental artifacts from constitutive promoters (62, 314, 315). Therefore, we recommend that any newly generated Cre driver line first be crossed with a reporter mouse (e.g., Rosa26-LSL-RFP/GFP/mCherry) to validate its specificity and recombination efficiency before being used in functional studies. Inducible systems such as Cre-ERT2 and Cre-DTR-mediated cell ablation systems are excellent choices for lineage tracing, studying spatiotemporal gene functions, or interrogating the physiological roles of specific cell types during atherosclerosis, as they enable highly selective and temporally controlled cell or gene ablation. However, Cre-ERT2 faces challenges including tamoxifen-induced metabolic perturbations and incomplete recombination (316–318), whereas Cre-DTR models have a short effective depletion window and risk off-target toxicity with extended dosing (319).

However, the successful use of constitutive Cre-loxP systems also hinges on the correct placement of loxP sites within the floxed allele. Improper intronic positioning-too close to splice junctions or within regulatory elements-can generate hypomorphic alleles that impair gene function even without Cre-mediated excision, potentially mimicking the knockout phenotype and complicating developmental studies (320). For inducible systems like Cre-ERT2, tamoxifen-independent leakiness is an equally critical concern; even without tamoxifen, basal Cre activity can cause premature recombination during development, precluding postnatal analysis of genes essential for early stages (318). Therefore, thorough validation of the floxed allele (e.g., phenotyping homozygous floxed mice in the absence of Cre) and assessment of baseline recombination in vehicle-treated controls are indispensable prerequisites for reliable phenotypic interpretation.

Emerging evidence supports the combined use of conventional knockout and Cre-loxP systems as a new paradigm in atherosclerosis research. On one hand, the development of knockout-first alleles allows the same targeted locus to be used either as a conventional null allele or as a conditional allele following Cre-mediated recombination (321). On the other hand, recent comparative analyses of conventional knockout, conditional knockout, and inducible systems underscore that these approaches are not mutually exclusive but rather complementary, with each offering distinct advantages for initial phenotypic screening vs. spatiotemporal functional dissection (4, 322). Notably, the increasing recognition of Cre toxicity and off-target effects further reinforces the need to benchmark conditional models against conventional knockouts whenever possible (323). Collectively, optimal model selection should be guided by specific research objectives: conventional knockouts excel in initial mechanistic screening and whole-body phenotype assessment, whereas Cre-loxP models are optimal for investigating spatiotemporal dynamics and cell-specific pathways (Table 10).

**Table 10:** Comparative analysis of conventional vs. Conditional vs. Inducible Mouse Models.

Dimension	Conventional Knockout Mice	Conditional Cre-loxP Mice	Inducible Cre-loxP Mice
Scope of Gene Ablation	Systemic, lifelong deletion	Cell/tissue-specific, temporally inducible	Cell/tissue-specific temporally restricted (postnatal/adultinduction)
Primary Research Applications	Basic gene function screening	Tissue-specific gene functions	Adult-onset disease modeling (e.g., cancer, neurodegeneration)
	Non-lethal systemic phenotypes	Functional studies of embryonic lethal genes	Lineage tracing and cell fate mapping
	Systemic genetic disease models	Stage-specific developmental functions	Distinguishing developmental vs. homeostatic functions
			Bypassing embryonic/neonatal lethality
Embryonic Lethality Issue	Cannot resolve (15%–30% genes cause lethality)	Circumventable (inducible ablation post-development)	Fully circumventable (induction at desired age, typically >4–6 weeks old)
Phenotype Interpretation	Challenging (confounding systemic effects)	Precise (localized phenotypic origin)	High spatiotemporal precision, but requires stringent controls to exclude
			Cre-recombinase toxicity and Tamoxifen off-target effects
Generation Timeline	Relatively short (∼6 months)	Extended (requires crossbreeding, ∼9–12 months)	Most extended (∼12–15 months, requires breeding for CreERT2 + floxed alleles, plus genotyping and induction validation)
Experimental VComplexity	Low	High (requires optimization of Cre efficiency/induction)	Highest (requires titration of Tamoxifen dose/route, leakiness assessment, and rigorous multi-layered controls for both Cre toxicity and Tamoxifen vehicle effects)

It is critical to note that the appropriate control for inducible Cre-loxP experiments is not simply the administration of tamoxifen to Cre-positive mice. Given the documented genotoxicity of Cre recombinase and the pleiotropic metabolic effects of tamoxifen, a minimum of three independent control cohorts are mandatory: (i) Cre-negative; Floxed/Floxed mice receiving tamoxifen to control for drug/vehicle effects, (ii) Cre-positive; Non-floxed (wild-type) mice receiving tamoxifen to control for Cre-off-target toxicity and (iii) Cre-positive; Floxed/Floxed mice receiving vehicle (oil) to assess basal Cre leakage. Adopting this three-tiered control strategy is critical for rigorously distinguishing bona fide conditional knockout phenotypes from nonspecific effects arising from Cre recombinase or tamoxifen administration (316, 324–326).

## Future directions

Although the Cre/loxP system has become the gold standard for cell-specific gene targeting in atherosclerosis research, existing models still suffer from several limitations, and no single ideal animal model exists for any particular clinical condition (307). These limitations include off-target recombination by Cre, developmental interference caused by constitutive promoters, and experimental variability introduced by conventional high-fat diet induction protocols. Future research should focus on the following directions.

First, the integration and upgrading of novel genetic tools. Dual-recombinase systems (e.g., combinations of Cre with Flp or Dre) enable precise targeting of specific cell subpopulations or spatiotemporally overlapping cell populations (327), overcoming the ambiguity in lineage tracing that arises from insufficient promoter specificity in single-Cre models. This approach has already proven valuable in dissecting smooth muscle cell lineages. For instance, a dual SMC-EC lineage-tracing model using the *Myh11^Dre^* and *Cdh5^Cre^* combination has revealed coordinated but functionally non-equivalent responses of smooth muscle and endothelial cells following irradiation, providing new insights into plaque stability (328). Similarly, an intersectional *Myh11^Dre^*; *Spp1^Cre^* system has enabled permanent labeling of SMC-derived cells with a history of endogenous Spp1 activation, facilitating the study of disease-associated SMC subpopulations (329). In addition, a recent study using the Gli1-CreER lineage-tracing tool successfully distinguished the genuine differentiation potential of adventitial Gli1⁺ stem cells from artifacts caused by ectopic Cre labeling, revealing that these cells contribute minimally to smooth muscle cells within atherosclerotic plaques (330). Beyond recombinase-based strategies, CRISPR/Cas9-mediated *in vivo* gene editing is fundamentally reshaping the disease modeling paradigm. Through AAV-delivered base editors or epigenetic editors such as CRISPR off, durable silencing of *PCSK9* or *ANGPTL3* can be achieved in the adult liver, producing sustained lipid-lowering and anti-atherosclerotic effects after a single administration, without altering the genomic DNA sequence and with significantly reduced off-target risk (331).

Second, the deep integration of multi-omics and dynamic phenotypic analysis. The combined application of single-cell sequencing, spatial transcriptomics, and lineage-tracing techniques now enables researchers to track the phenotypic transition trajectories of specific cell lineages during plaque progression at single-cell resolution (331). In the future, integrating these phenotypic data with Cre-mediated gene-function interventions will facilitate the systematic construction of “fate maps” for all major cell types involved in atherosclerosis, thereby elucidating the temporal dynamics of cell-state transitions and their contributions to disease outcomes.

In summary, the next generation of mouse models for atherosclerosis should achieve systematic breakthroughs in genetic precision, spatiotemporal controllability, human relevance, and multidimensional phenotypic analysis, thereby bridging the gap between fundamental mechanistic insights and clinical translation.

## Conclusion

Cre mouse models are essential for dissecting atherosclerosis mechanisms and accelerating therapeutic discovery, and careful selection is critical because different models may yield divergent effects on lesion development. Future efforts would benefit from prioritizing human-relevant technologies including single-cell RNA sequencing, dual-recombinase, lineage tracing, and comorbidity modeling, along with standardized protocols and cross-species validation to bridge translational gaps.

## Glossary

CVD: cardiovascular disease
CRISPR: clustered regularly interspaced short palindromic repeats
Cre: cyclization recombination
loxP: locus of X-over P1
APOE: apolipoprotein E
LDLR: low density lipoprotein receptor
VLDL: very low density lipoprotein
AAV: adeno-associated virus
LDL: low density lipoprotein
HDL: high density lipoprotein
PCSK9: proprotein convertase subtilisin/kexin type 9
T2D: type 2 diabetes
NK cell: natural killer cell
Tie2-Cre: tie2 promoter-driven cre recombinase
Cdh5-Cre: cadherin 5 promoter-driven cre recombinase
FLK1: fetal liver kinase 1
Pdgfb: platelet-derived growth factor subunit B
EC: endothelial cell
SMC: smooth muscle cell
Lyve1: lymphatic vessel endothelial hyaluronan receptor 1
LEC: lymphatic endothelial cell
LSEC: liver sinusoidal endothelial cell
BEC: blood endothelial cell
Prox1: prospero homeobox protein 1
VSMC: vascular smooth muscle cell
SM22α: smooth muscle 22 Alpha
AKT1: AKT serine/threonine kinase 1
PTEN: phosphatase and tensin homolog
FOXO1/3: forkhead box O1 and O3
Runx2: runt-related transcription factor 2
SCAP: SREBP cleavage-activating protein
Nedd4: neural precursor cell expressed developmentally down-regulated 4
ROS: reactive oxygen species
AMPK: AMP-activated protein kinase
MT1-MMP: membrane type 1 matrix metalloproteinase
NF-κB: nuclear factor kappa-light-chain-enhancer of activated B cells
Myh11: myosin heavy chain 11
KLF: krüppel like factor
Itga8: integrin subunit alpha 8
Lyz2: lysozyme 2
Acta2: Actin Alpha 2, smooth muscle
DC: dendritic cell
LysM: lysosomal membrane protein
CX3CR1: CX3C chemokine receptor 1
Ly6C: lymphocyte antigen 6 complex, locus C
Mafb: MAF bZIP transcription factor B
DTR: diphtheria toxin receptor
CCR2: C-C chemokine receptor type 2
RFP: red fluorescent protein
PR3: proteinase 3
NE: neutrophil elastase
NETs: neutrophil extracellular traps
LY6G: lymphocyte antigen 6 complex, Locus G
cDCs: conventional DCs
cDC1s: conventional type 1 dendritic cells
Zbtb46: Zinc finger and BTB domain containing 46
GM-CSF: granulocyte-macrophage colony-stimulating factor
DTA: diphtheria toxin A
Xcr1: X-C motif chemokine receptor 1
Clec9a: C-type lectin domain family 9 member A
IFN-γ: interferon-gamma
TNF-α: tumor necrosis factor-alpha
Tregs: regulatory T cells
Th1: T helper 1
LCK: lymphocyte-specific protein tyrosine kinase
TCR: T cell receptor
TGF-β: transforming growth factor-beta
IL-4: interleukin-4
Foxp3: forkhead box P3
RORγt: retinoid-related orphan receptor gamma t
NK cells: natural killer cells
NOD-SCID: non-obese diabetic-severe combined immunodeficiency
NOG: NOD/Shi-scid/IL-2Rγnull
μMT: μ heavy chain membrane exon targeted
Col1a1: collagen type i alpha 1 chain
ECM: extracellular matrix
Tcf21: transcription factor 21
Postn: periostin
PDGFRα: platelet-derived growth factor receptor alpha
Alb: albumin
TBG: thyroxine binding globulin
Adipoq: adiponectin
Cas9: CRISPR-associated protein 9
CETP: cholesteryl ester transfer protein.
